# Supplementary material for: Sex and APOE ε2 Interactive Effects on the Longitudinal Change in Cognition in a Population-Based Cohort of Older Adults with Vascular Risk Factors
Source: Int J Mol Sci. 2025 Oct 30;26(21):10591. doi: 10.3390/ijms262110591 (PMC12607810; doi:10.3390/ijms262110591)
Supplement: Supplementary file 1 [file ijms-26-10591-s001.zip › ijms-3707251-supplementary.pdf]

Supplementary data

SUPPLEMENTARY TABLE S1

Supplementary Table S1. Sociodemographic, clinical and neuropsychological sample characteristics of the subjects who dropped out of the study.

	**Total (n =260)**	**Men (n=178)**	**Women (n = 82)**
**Sociodemographic and clinical data**			
Age (years)	68.10 (8.48)	69.4 (8.46)	65.37 (7.90)
Sex (% women)	31.50 (82)	-	-
Education (years)	5.86 (4.05)	6.21 (4.18)	5.10 (3.65)
GDS-15	2.40 (2.40)	1.80 (1.57)	3.70 (3.26)
REGICOR	8.83 (4.81)	10.03 (5.16)	6.17 (2.30)
ε2 vs non-ε2 (% ε2 carriers (n))	10.00 (26)	9.00 (16)	12.2 (10)
ε4 vs non-ε4 (% ε4 carriers (n))	21.5 (56)	21.30 (38)	22.00 (18)
**Neuropsychological domains (Z scores)**			
Visuospatial skills/speed	-0.06 (0.41)	-0.04 (0.39)	-0.13 (0.41)
Verbal memory	-0.17 (0.88)	-0.16 (0.39)	-0.14 (0,92)
Verbal fluency	-0.20 (0.83)	-0.08 (0.83)	-0.40 (0.83)

Note: Continuous variables are reported as mean (SD).

SUPPLEMENTARY TABLE S2

Supplementary Table S2. Linear regression results for association between *APOE* alleles and Z-scores at baseline for cognitive domains only in participants that continued to follow-up.

	**Visuospatial skills/speed**					**Verbal memory**					**Verbal fluency**				
**TOTAL**	*n*	*β*	*95% CI*	*p*	*p-perm*	*n*	*β*	*95% CI*	*p*	*p-perm*	*n*	*β*	*95% CI*	*p*	*p-perm*
APOE ε4	375	0.150	-0.019/0.320	0.082	0.274	375	-0.062	-0.282/0.157	0.578	0.955	375	-0.014	-0.212/0.183	0.888	1
APOE ε2	375	-0.185	-0.415/0.045	0.116	0.373	375	0.378	0.081/0.674	**0.013**	**0.050**	375	0.295	0.029/0.562	**0.030**	0.106
**MEN**	*n*	*β*	*95% CI*	*p*	*p-perm*	*n*	*β*	*95% CI*	*p*	*p-perm*	*n*	*β*	*95% CI*	*p*	*p-perm*
APOE ε4	234	0.125	-0.073/0.323	0.217	0.566	234	-0.045	-0.305/0.214	0.732	0.995	234	-0.038	-0.288/0.212	0.766	0.996
APOE ε2	234	-0.340	-0.643/-0.037	**0.029**	0.114	234	0.043	-0.358/0.443	0.835	1	234	0.223	-0.162/0.608	0.257	0.645
**WOMEN**	*n*	*β*	*95% CI*	*p*	*p-perm*	*n*	*β*	*95% CI*	*p*	*p-perm*	*n*	*β*	*95% CI*	*p*	*p-perm*
APOE ε4	141	0.197	-0.140/0.534	0.254	0.692	141	-0.108	-0.541/0.326	0.627	0.980	141	0.035	-0.299/0.370	0.836	1
APOE ε2	141	0.095	-0.268/0.458	0.608	0.980	141	0.855	0.413/1.298	**<0.001**	**0.002**	141	0.436	0.085/0.788	**0.016**	0.075

Note: β coefficients (and 95%CI) represent the effect of each extra minor allele. Age, sex, years of schooling, baseline depression, and baseline REGICOR have been included as covariates in all analyses. Analyses have been run under an additive (i.e. allele-dose dependent) genetic model. *p-perm*: probability of the observed p-values after 1000 permutations.

SUPPLEMENTARY TABLE S3

Supplementary Table S3. Linear regression results for association between *APOE* alleles and Z-scores at baseline for cognitive domains only in participants that did not continue to follow-up.

	Visuospatial skills/speed					Verbal memory					Verbal fluency				
**TOTAL**	*n*	*β*	*95% CI*	*p*	*p-perm*	*n*	*β*	*95% CI*	*p*	*p-perm*	*n*	*β*	*95% CI*	*p*	*p-perm*
APOE ε4	257	0.190	-0.025/0.405	0.085	0.296	257	-0.152	-0.384/0.079	0.198	0.556	257	0.149	-0.080/0.377	0.204	0.596
APOE ε2	257	-0.037	-0.347/0.274	0.816	1	257	0.327	-0.005/0.658	0.054	0.191	257	0.425	0.099/0.750	**0.011**	0.055
**MEN**	*n*	*β*	*95% CI*	*p*	*p-perm*	*n*	*β*	*95% CI*	*p*	*p-perm*	*n*	*β*	*95% CI*	*p*	*p-perm*
APOE ε4	177	0.274	-0.001/0.549	0.052	0.206	177	-0.115	-0.420/0.190	0.460	0.909	177	0.184	-0.114/0.482	0.228	0.640
APOE ε2	177	0.049	-0.343/0.441	0.807	0.999	177	0.168	-0.261/0.598	0.443	0.896	177	0.223	-0.197/0.643	0.300	0.757
**WOMEN**	*n*	*β*	*95% CI*	*p*	*p-perm*	*n*	*β*	*95% CI*	*p*	*p-perm*	*n*	*β*	*95% CI*	*p*	*p-perm*
APOE ε4	80	0.064	-0.286/0.415	0.720	0.996	80	-0.225	-0.585/0.135	0.224	0.625	80	0.112	-0.257/0.480	0.554	0.958
APOE ε2	80	-0.258	-0.779/0.264	0.336	0.760	80	0.539	0.010/1.068	**0.049**	0.175	80	0.712	0.185/1.240	**0.010**	**0.042**

Note: β coefficients (and 95%CI) represent the effect of each extra minor allele. Age, sex, years of schooling, baseline depression, and baseline REGICOR have been included as covariates in all analyses. Analyses have been run under an additive (i.e. allele-dose dependent) genetic model. *p-perm*: probability of the observed p-values after 1000 permutations.

SUPPLEMENTARY TABLE S4

Supplementary Table S4. Comparison of different adjustments on the longitudinal association analyses for the verbal memory domain.

	Baseline-adjusted					Adjusted for mean follow-up/baseline Z-score					No baseline adjustment				
**TOTAL**	*n*	*β*	*95% CI*	*p*	*p-perm*	*n*	*β*	*95% CI*	*p*	*p-perm*	*n*	*β*	*95% CI*	*p*	*p-perm*
APOE ε4	374	-0.147	-0.320/0.027	0.098	0.320	374	-0.127	-0.328/0.075	0.218	0.591	374	-0.119	-0.320/0.083	0.249	0.642
APOE ε2	374	-0.238	-0.474/-0.001	**0.049**	0.180	374	-0.401	-0.671/-0.131	**0.004**	**0.019**	374	-0.411	-0.680/-0.141	**0.003**	**0.015**
**MEN**	*n*	*β*	*95% CI*	*p*	*p-perm*	*n*	*β*	*95% CI*	*p*	*p-perm*	*n*	*β*	*95% CI*	*p*	*p-perm*
APOE ε4	233	-0.163	-0.352/0.026	0.093	0.270	233	-0.157	-0.380/0.066	0.170	0.285	233	-0.141	-0.366/0.083	0.218	0.562
APOE ε2	233	-0.382	-0.670/-0.095	**0.010**	**0.027**	233	-0.429	-0.768/-0.089	**0.014**	**0.033**	233	-0.408	-0.749/-0.067	**0.020**	0.058
**WOMEN**	*n*	*β*	*95% CI*	*p*	*p-perm*	*n*	*β*	*95% CI*	*p*	*p-perm*	*n*	*β*	*95% CI*	*p*	*p-perm*
APOE ε4	141	-0.116	-0.499/0.266	0.552	0.961	141	-0.069	-0.502/0.364	0.756	0.999	141	-0.073	-0.050/0.357	0.739	0.994
APOE ε2	141	-0.017	-0.448/0.414	0.939	1	141	-0.461	-0.935/0.013	0.059	0.2218	141	-0.406	-0.864/0.051	0.084	0.285

Note: results for adjustments by baseline scores, mean between baseline and follow-up scores, and no baseline adjustment are shown. β coefficients (and 95%CI) represent the effect of each extra minor allele. Age, sex, years of schooling, baseline depression, and baseline REGICOR have been included as covariates in all analyses. Analyses have been run under an additive (i.e. allele-dose dependent) genetic model. *p-perm*: probability of the observed p-values after 1000 permutations.

SUPPLEMENTARY TABLE S5

Supplementary Table S5. Age-stratified association analyses between *APOE* alleles and changes in verbal memory from baseline to follow-up.

	Younger (≤70 years old)					Older (>70 years old)				
**TOTAL**	*n*	*β*	*95% CI*	*p*	*p-perm*	*n*	*β*	*95% CI*	*p*	*p-perm*
APOE ε4	179	-0.160	-0.420/-0.101	0.232	0.575	195	-0.132	-0.369/0.105	0.277	0.692
APOE ε2	179	-0.245	-0.578/0.089	0.152	0.424	195	-0.241	-0.602/0.120	0.193	0.545
**MEN**	*n*	*β*	*95% CI*	*p*	*p-perm*	*n*	*β*	*95% CI*	*p*	*p-perm*
APOE ε4	92	-0.094	-0.411/0.222	0.559	0.948	141	-0.225	-0.464/0.014	0.067	0.223
APOE ε2	92	-0.619	-1.085/-0.153	**0.011**	**0.047**	141	-0.197	-0.587/0.193	0.324	0.739
**WOMEN**	*n*	*β*	*95% CI*	*p*	*p-perm*	*n*	*β*	*95% CI*	*p*	*p-perm*
APOE ε4	87	-0.327	-0.816/0.161	0.193	0.553	54	0.267	-0.350/0.883	0.401	0.897
APOE ε2	87	0.171	-0.327/0.668	0.503	0.921	54	-0.342	-1.215/0.531	0.447	0.922

Note: β coefficients (and 95%CI) represent the effect of each extra minor allele. Sex, years of schooling, baseline depression, baseline REGICOR and baseline domain Z-score have been included as covariates in all analyses. Analyses have been run under an additive (i.e. allele-dose dependent) genetic model. *p-perm*: probability of the observed p-values after 1000 permutations.

SUPPLEMENTARY TABLE S6

Supplementary Table S6. Gene-gene interaction effects on cognitive change.

	**Visuospatial skills/speed**		**Verbal memory**		**Verbal fluency**	
	β	*p*	*β*	*p*	*β*	*p*
BDNF x ε2	1.231	0.097	0.435	0.459	1.041	0.066
BDNF x ε4	-0.203	0.590	-0.583	0.054	-0.251	0.390

SUPPLEMENTARY TABLE S7

Supplementary Table S7. Description of cognitive domains and neuropsychological tests administered.

**Factors and tests**	**Cognitive ability tested**	**Raw scores**
Visuospatial skills/speed		
Visual reproduction-immediate recall	Immediate visual memory	
(WMS-III)	Delayed visual memory	
Visual reproduction-delayed recall	Visoconstructive abilities	
(WMS-III)	Attention, working memory	
Visual reproduction-copy (WMS-III)	Psychomotor speed, visuomotor	
Digit Symbol Coding (WAIS-III)	coordination	
Grooved Pegboard Test (preferred hand)	Attention, visual scanning, psychomotor	
Trail Making Test Part A	speed	
Verbal memory		
Word list-immediate recall (WMS-III)	Immediate verbal memory	
Word list-delayed recall (WMS-III)	Delayed verbal memory	
Verbal fluency		
Letter fluency (P)	Language, executive functions	
Semantic fluency (animals)	Language, executive functions	

Note: values are means (SD). The Trail Making Test and Grooved Pegboard Test results are expressed in seconds. WMS: Wechsler Memory Scale; WAIS: Wechsler Adult Intelligence Scale.
